# Patients With Critical Humeral Bone Loss Have Similar Patient‐Reported Outcomes to Patients Without Critical Humeral Bone Loss After Arthroscopic Shoulder Stabilization at a Mean Follow‐Up of 7 Years

**DOI:** 10.1002/ars2.70140

**Published:** 2026-11-03

**Authors:** Ryan Gilbert, Ryan T. Lin, Sahil Dadoo, Andrew Liu, Ting Cong, Adam Popchak, Bryson P. Lesniak, Mark Rodosky, Jonathan D. Hughes, Albert Lin

**Affiliations:** ^1^ University of Pittsburgh School of Medicine Pittsburgh Pennsylvania U.S.A.; ^2^ Department of Orthopaedic Surgery University of Pittsburgh Medical Center Pittsburgh Pennsylvania U.S.A.; ^3^ Sutter Health, Orthopedics and Sports Medicine Sacramento California U.S.A.

## Abstract

**Purpose:**

To compare long‐term patient‐reported outcomes (PROs) between patients with and without critical humeral bone loss (CHBL) and to compare long‐term PROs among patients with CHBL who underwent arthroscopic Bankart repair (ABR) alone versus those who received a remplissage augmentation (ABR + R).

**Methods:**

A retrospective review included patients aged 13 to 50 years who underwent ABR or ABR + R for anterior shoulder instability from 2007 to 2023. Exclusion criteria included <1‐year follow‐up, >20% glenoid bone loss, off‐track Hill‐Sachs lesion, concomitant pathology, or revision surgery. CHBL was defined as humeral head defect extension below the 90° equator. Western Ontario Shoulder Index, pain Visual Analog Scale, and Subjective Shoulder Value were collected at the final follow‐up. PROs and recurrence rates were compared between cohorts and within the CHBL subgroup.

**Results:**

Three hundred eighty‐four patients were included (mean age 23 ± 9 years (range: 13‐49), 78% men), of which 99 (26%) were successfully contacted for PROs: 129 (34%) with CHBL and 255 (66%) without CHBL. The CHBL cohort was older (25 vs 22 years, *P* < .01) and had smaller distance to dislocation (9 vs 13 mm, *P* < .01). Recurrent instability rates were comparable overall (22% vs 15%, *P* = .08), but, within the CHBL cohort, recurrence was significantly lower after ABR + R than ABR alone (9% vs 32%, *P* < .01). Ninety‐nine patients (26%) (41 CHBL, 58 non‐CHBL) completed PROs at a mean follow‐up of 7.3 years (range: 1.9‐14.4), with no significant differences in Western Ontario Shoulder Index, pain Visual Analog Scale, Subjective Shoulder Value, or recurrent instability between groups (all *P* > .05). In a multivariable linear regression, only recurrent instability was an independent predictor of inferior PROs; the presence of CHBL was not an independent predictor. Among CHBL patients, PROs were similar between ABR and ABR + R.

**Conclusions:**

Patients with CHBL had similar long‐term PROs to those without CHBL after ABR or ABR + R at a minimum follow‐up of 1 year. Furthermore, the addition of remplissage was found to be associated with a reduced rate of recurrent anterior shoulder instability in patients with CHBL.

**Level of Evidence:**

Level III, retrospective cohort study.

## Graphical abstract

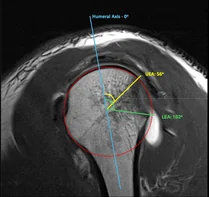

Anterior shoulder dislocations are often complicated by traumatic bony defects to the glenoid and/or humeral head that further compromise shoulder stability. Rates of recurrent anterior shoulder instability after arthroscopic Bankart repair (ABR) are reported as high as 30% in high‐risk populations, especially among contact athletes and younger patients.^1^, ^2^, ^3^ Engaging, or “off‐track,” Hill‐Sachs lesions (HSLs) of the humeral head have emerged as independent predictors of recurrent anterior shoulder instability, with the addition of remplissage offering protection against recurrent anterior shoulder instability in the setting of “off‐track” HSLs.^4^, ^5^, ^6^

Recent literature suggests that HSL characteristics outside the concept of “on‐track” versus “off‐track” play an important role in the risk of HSL engagement and recurrent anterior shoulder instability after ABR. Cong et al. described critical humeral bone loss (CHBL) as an HSL extending inferiorly below the humeral head equator (≥90° on sagittal magnetic resonance imaging [MRI]) and found CHBL to be an independent predictor of recurrent anterior shoulder instability after ABR.^7^ Subsequent analysis by Steuer et al. and Honoki et al. showed that the addition of remplissage (ABR + R) in patients with CHBL significantly decreased recurrent anterior shoulder instability rates.^8^, ^9^ These data suggest that CHBL plays an important role in the context of recurrent anterior shoulder instability after arthroscopic shoulder stabilization and that remplissage serves as an effective augmentation to mitigate recurrent anterior shoulder instability in patients with inferior HSL extension.

However, it remains unclear how CHBL may influence patient‐reported outcomes (PROs) after arthroscopic surgical stabilization. The purposes of this study were to compare long‐term PROs between patients with and without CHBL and to compare long‐term PROs among patients with CHBL who underwent ABR versus ABR + R. We hypothesized that patients with CHBL would have worse PROs at long‐term follow‐up compared with patients without CHBL and that patients with CHBL who underwent ABR + R would have superior PROs compared with patients with CHBL who underwent ABR alone.

## METHODS

### Study Population

Institutional Review Board approval was obtained from the University of Pittsburgh (No. STUDY20030061). A retrospective review was conducted of consecutive patients with shoulder instability who underwent surgical stabilization between 2007 and 2023 by 1 of 6 fellowship‐trained orthopaedic sports medicine surgeons within a single institution.

All patients who underwent arthroscopic repair of the labrum with at least 3 anchors in the context of anterior shoulder instability between 2007 and 2023 were identified. First, all cases of nonanterior stabilization (isolated repair of SLAP tears or repair of circumferential labral tears) were excluded. Next, patients were excluded for a history of uncontrolled seizure disorder, connective tissue disease, posterior or multidirectional instability, a prior history of ipsilateral shoulder surgery not including stabilization, concomitant rotator cuff repair, concomitant glenoid fracture repair, or open stabilization surgery. Of this remaining cohort, study‐specific exclusion criteria were applied which included less than 1 year of follow‐up, revision stabilization surgery, an off‐track HSL,^10^ glenoid bone loss (GBL) greater than 20%, age under 13 or over 50 years, an absent HSL on MRI, and missing or inaccessible MRI. Patients were categorized by the presence or absence of CHBL based on the measurement technique described below, as well as whether they underwent ABR or ABR + R.

### Variable Collection

Extracted variables included demographics (age and sex), sport type (contact and/or collision), glenohumeral capsuloligamentous laxity, and surgery type (ABR vs ABR + R). Contact and collision sport classifications were not mutually exclusive. Collision sports included football, lacrosse, hockey, and wrestling. Contact sports included all collision sports as well as soccer and basketball. Sports that were neither contact nor collision included swimming, baseball, volleyball, track and field, skiing, and dance.

Preoperative glenohumeral hyperlaxity was determined during examination under anesthesia, as described by Boden et al.^11^ Glenohumeral hyperlaxity was defined as external rotation >85° with the arm at the side and/or grade 2 or 3 humeral head translation relative to the glenoid during anterior, posterior, or inferior load‐and‐shift testing at 90° abduction. Grade 2 translation was defined as humeral head translation over the glenoid rim with spontaneous reduction, and grade 3 translation was defined as humeral head translation without spontaneous reduction.^12^

All eligible patients were contacted by telephone to complete a questionnaire assessing postoperative PROs. After informed consent was obtained, PROs were assessed using the Western Ontario Shoulder Instability Index (WOSI),^13^ pain Visual Analog Scale (pVAS),^14^ and Subjective Shoulder Value (SSV).^15^ The WOSI is comprised of 4 subdomains with individuals scores totaling to 2100: physical symptoms (0‐1000), sports/recreation/work impact (0‐400), lifestyle impact (0‐400), and emotional impact (0‐300). Higher values on the WOSI and pVAS scales indicate lower shoulder function, whereas lower values on SSV indicate lower shoulder function. A recurrence of anterior shoulder instability was determined by review of the electronic medical record (EMR) and defined as recurrent glenohumeral subluxation or dislocation because of the index surgery. Subluxation was defined as a self‐reported sensation of instability or apprehension; dislocation was defined as requiring manual reduction. Although recurrent instability data were also collected via telephone interview for patients who completed PRO follow‐up, recurrence was determined by EMR for the full cohort of patients to maintain a consistent data source across all included patients. The primary outcome was raw numerical score for WOSI. Secondary outcomes were raw numerical scores for pVAS and SSV, as well as the rate of recurrent anterior shoulder instability.

### MRI Measurements

All MRI measurements were obtained by an orthopaedic surgery fellow, resident, and medical student (R.G., T.C., and M.J.) with extensive experience in shoulder MRI measurements. All raters were trained, and inter‐rater reliability was confirmed prior to image analysis.

Anatomic measurements evaluated on preoperative MRI included GBL, Hill‐Sachs interval (HSI), distance to dislocation (DTD), and craniocaudal Hill‐Sachs extension. GBL was measured as a percentage of anterior GBL relative to the total glenoid width using the perfect circle method on T2‐weighted sagittal MRI sequences.^16^ The HSI was measured on axial, T2‐weighted MRI sequences as the distance from the medial‐most aspect of the HSL to the infraspinatus insertion site laterally. The glenoid track (GT) was measured by the method described by Yamamoto et al.^10^ using the formula GT* = *0.83 * D − d, wherein *D* is the diameter of the glenoid best‐fit circle and *d* is the width of anterior GBL. DTD could then be calculated with the HSI and GT using the formula DTD = GT *−* HSI.^17^ A negative DTD was considered “off‐track” and therefore excluded from the study.

Finally, we used the method described by Cong et al.^7^ to evaluate the craniocaudal characteristics of the HSL and define CHBL. Measurements were performed on the sagittal MRI slice with the largest craniocaudal length of the HSL. A best‐fit circle was first drawn over the humeral head. A line was then drawn along the humeral shaft axis through the center of the best‐fit circle. The HSL position was measured by the upper edge angle and lower edge angle relative to the humeral axis. Based on work by Cong et al., a lower edge angle extending below 90° (with 0° being defined as 12 o'clock on the best fit circle) constitutes CHBL (Figure 1).

**FIGURE 1:**
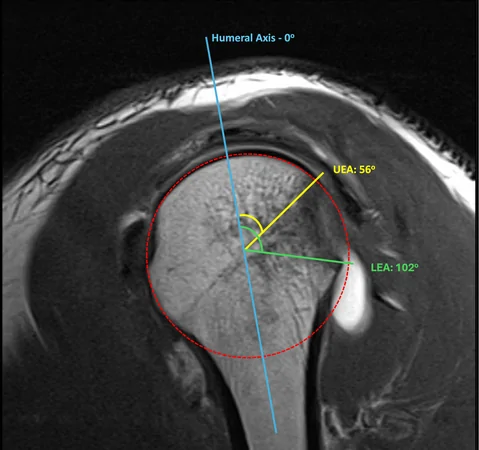
Hill‐Sachs craniocaudal grading showing critical humeral bone loss. The sagittal MRI slice with the largest craniocaudal length of the HSL is selected. A best‐fit circle (red) is drawn over the humeral head. A line (blue) is drawn through the center of the humeral shaft axis bisecting the best fit circle. Two lines each bisecting the best fit circle are then drawn from the upper (yellow) and lower (green) limits of the HSL. These lines form UEA and LEA of the HSL. The angle is calculated from the 12 o'clock position of the humeral axis, defined as 0°. A LEA extending below 90° is considered critical humeral bone loss. (HSL, Hill‐Sachs lesion; LEA, lower edge angle; MRI, magnetic resonance imaging; UEA, upper edge angle.)

### Surgical Technique

All procedures were performed in either the lateral decubitus or beach‐chair position, according to the surgeon preference. Anterior labral repair was performed arthroscopically using a standard suture anchor technique with a minimum of 3 anchors. All remplissage augmentations were performed using 2 anchors to secure the posterior capsule with a double‐pulley method, as described by Nazzal et al.^18^ At our institution in the time frame from which these patients were queried, remplissage was primarily indicated for patients with “off‐track” HSLs and <20% GBL, “on‐track” or “near‐track” lesions (DTD < 10 mm), or consideration of additional risk factors such as contact athletics, glenohumeral hyperlaxity, and/or age <25 years.

### Statistical Analysis

Statistical analyses were performed in Stata v.18.5 (StataCorp, College Station, TX). Continuous data were assessed for normality via Q‐Q plots and compared using appropriate parametric or nonparametric tests. Categorical variables were analyzed with *χ* ^2^ or Fisher's exact tests. Demographic characteristics, postoperative PROs, and rates of recurrent anterior shoulder instability were compared between patients with and without CHBL. A multivariable linear regression was performed to determine the independent association of CHBL with postoperative PROs. Models were adjusted for age, sex, DTD, recurrent instability, remplissage augmentation, revision surgery, and follow‐up time. A subgroup analysis was performed among patients with CHBL comparing PROs after ABR versus ABR + R. Statistical significance was set at *P* < .05.

We conducted a power analysis based on the work of Park et al., which established a minimal clinically important difference (MCID) for the WOSI score after ABR of 151.9.^19^ To detect a difference equal to half of the MCID (76.0) between patients with and without CHBL, with 80% power and a two‐sided alpha of 5%, we calculated a required sample size of 54 patients per cohort.

## RESULTS

### Cohort Overview

Three hundred eighty‐four patients (mean age 23 ± 9 years (range: 13‐49); 78% men) met inclusion criteria (Figure 2); 99 (26%) were successfully contacted for PROs. One hundred twenty‐nine patients (34%) had CHBL, and 255 (66%) did not have CHBL (non‐CHBL). Ninety‐nine patients (26%) were contacted for PROs at a mean follow‐up of 7.3 ± 3.9 (range: 1.9‐14.4) years, with 41 (42%) having CHBL and 58 (59%) not having CHBL. Cohort demographics and anatomical characteristics are summarized in Table 1.

**FIGURE 2:**
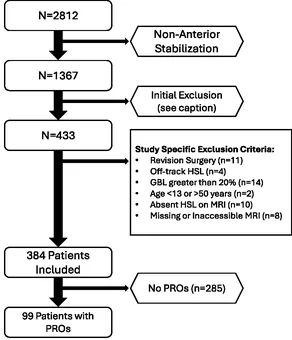
Exclusion flowchart. All patients who underwent arthroscopic stabilization surgery for shoulder instability between 2007 and 2023 were identified. First, all cases of nonanterior stabilization were excluded. Next, patients were excluded for a history of uncontrolled seizure disorder, connective tissue disease, posterior or multidirectional instability, a prior history of ipsilateral shoulder surgery not including stabilization, concomitant rotator cuff repair, concomitant glenoid fracture repair, or open stabilization surgery. Of this cohort, study‐specific exclusion criteria were applied which included less than 1 year of follow‐up, revision stabilization surgery, an off‐track HSLs, glenoid bone loss greater than 20%, patient age under 13 or over 50 years, an absent HSL on MRI, missing or inaccessible MRI, and missing postoperative survey data. (GBL, glenoid bone loss; HSL, Hill‐Sachs lesion; MRI, magnetic resonance image; PROs, patient‐reported outcomes.)

**TABLE 1:** Cohort Demographics

	**Total (n = 384)**	**CHBL (n = 129)**	**Non‐CHBL (n = 255)**	***P* Value**
Age at surgery, yr (mean ± SD)	23 ± 9	25 ± 10	22 ± 8	**<.01**
Male sex, no. (%)	300 (78)	101 (78)	199 (78)	.96
Contact athlete, no. (%)	165 (43)	53 (41)	112 (44)	.60
Glenohumeral hyperlaxity, no. (%)	41 (11)	18 (14)	23 (9)	.14
% Glenoid bone loss (mean ± SD)	8 ± 11	9 ± 10	7 ± 11	.08
DTD, mm (mean ± SD)	12 ± 6	9 ± 4	13 ± 6	**<.01**
Procedure, no. (%)				.07
ABR	238 (62)	72 (56)	166 (65)	
ABR + R	146 (38)	57 (44)	89 (35)	

*Note*: Data are presented as mean ± SD for continuous measures, and no. (%) for categorical measures. Bolded values indicate statistical significance (*P* < .05).

ABR, arthroscopic Bankart repair; ABR + R, arthroscopic Bankart repair with remplissage; CHBL, critical humeral bone loss; DTD, distance to dislocation; SD, standard deviation; yr, years.

Patients with CHBL were older than those without CHBL (25 vs 22 years, *P* < .01). There were no significant differences between the CHBL and non‐CHBL cohorts with regard to participation in contact athletics (41% vs 44%, *P* = .60), the presence of glenohumeral hyperlaxity (14% vs 9%, *P* = .14), or mean percent GBL (9% vs 7%, *P* = .08). The CHBL cohort had a smaller mean DTD (9 mm vs 13 mm, *P* < .01) than the non‐CHBL cohort. Rates of remplissage augmentation did not significantly differ between CHBL and non‐CHBL cohorts (44% vs 35%, *P* = .07).

### Patient‐Reported Outcomes

Among the 99 of 384 patients for whom PROs were available, no significant differences were observed between CHBL and non‐CHBL cohorts in WOSI (278 ± 395 vs 347 ± 415, *P* = .41), pVAS (1 ± 2 vs 2 ± 2, *P* = .35), nor SSV (79 ± 20 vs 84 ± 18, *P* = .18). Analysis of WOSI subdomains revealed no significant differences between CHBL and non‐CHBL cohorts (all *P* > .05) (Table 2; Supporting Information). Demographics of the 99 patients successfully contacted for PROs are shown in the Table S1.

**TABLE 2:** Patient‐Reported Outcomes, Stratified by Critical Humeral Bone Loss

	**Total**	**CHBL**	**No CHBL**	***P* Value**
	**(n = 99)**	**(n = 41)**	**(n = 58)**	
Follow‐up time, yr (mean ± SD)	7.3 ± 3.9	7.0 ± 3.8	7.6 ± 4.1	.42
WOSI score (mean ± SD)	319 ± 406	278 ± 395	347 ± 415	.41
Physical symptoms subscore	148 ± 194	113 ± 179	172 ± 201	.14
Sports/rec/work subscore	57 ± 85	49 ± 83	61 ± 87	.50
Lifestyle subscore	48 ± 78	49 ± 83	47 ± 75	.97
Emotional subscore	68 ± 79	69 ± 77	67 ± 81	.93
pVAS (mean ± SD)	2 ± 2	1 ± 2	2 ± 2	.35
SSV (mean ± SD)	82 ± 19	79 ± 20	84 ± 18	.18

*Note*: Data are presented as mean ± SD. Data are significant if *P* < .05.

CHBL, critical humeral bone loss; pVAS, pain Visual Analog Scale; SD, standard deviation; SSV, Subjective Shoulder Value; WOSI, Western Ontario Shoulder Instability Index; yr, years.

In a subgroup analysis of the CHBL cohort in whom PROs were available, ABR + R did not lead to significantly better PROs compared with ABR. Patients who underwent ABR versus ABR + R had comparable WOSI (347 ± 503 vs 225 ± 285, *P* = .33), pVAS (2 ± 3 vs 1 ± 2, *P* = .20), and SSV (76 ± 22 vs 82 ± 18, *P* = .39) scores, with no significant differences in any WOSI subdomains observed between patients undergoing ABR versus ABR + R (all *P* > .05; Table 3).

**TABLE 3:** Patient‐Reported Outcomes in Patients With Critical Humeral Bone Loss, Stratified by Remplissage Augmentation

	**Total**	**ABR**	**ABR + R**	***P* Value**
	**(n = 41)**	**(n = 23)**	**(n = 18)**	
WOSI score (mean ± SD)	278 ± 395	225 ± 285	347 ± 503	.33
Physical symptoms subscore	113 ± 179	91 ± 122	142 ± 234	.37
Sports/rec/work subscore	49 ± 83	39 ± 71	62 ± 96	.38
Lifestyle subscore	48 ± 82	34 ± 59	66 ± 105	.22
Emotional subscore	69 ± 77	63 ± 65	76 ± 92	.60
pVAS (mean ± SD)	1 ± 2	1 ± 2	2 ± 3	.20
SSV (mean ± SD)	79 ± 20	82 ± 18	76 ± 22	.39

*Note*: Data are presented as mean ± SD. Data are significant if *P* < .05.

ABR, arthroscopic Bankart repair; ABR + R, arthroscopic Bankart repair with remplissage; pVAS, pain Visual Analog Scale; SD, standard deviation; SSV, Subjective Shoulder Value; WOSI, Western Ontario Shoulder Instability Index.

### Recurrent Anterior Shoulder Instability

Sixty‐five (17%) patients sustained recurrent anterior shoulder instability, and 25 (7%) underwent revision stabilization (Table 4). Rates of recurrent anterior shoulder instability did not differ significantly between the CHBL and non‐CHBL cohorts (28/129 [22%] vs 37/255 [15%], *P* = .08), nor did rates of revision surgery. Among the CHBL cohort, however, rates of recurrent anterior shoulder instability were significantly lower for patients who underwent ABR + R compared with patients who underwent ABR alone (5/57 [9%] vs 23/72 [32%], *P* < .01), as was the rate of revision surgery.

**TABLE 4:** Rates of Recurrent Anterior Shoulder Instability and Revision Surgery

	**Recurrent Instability**	***P* Value**	**Revision Surgery**	***P* Value**
Full cohort (n = 384), no. (%)	65 (17)	.08	25 (7)	.48
No CHBL (n = 255)	37 (15)		15 (6)	
CHBL (n = 129)	28 (22)		10 (8)	
CHBL cohort (n = 129), no. (%)	28 (22)	**<.01**	10 (8)	**.02**
ABR (n = 72)	23 (32)		9 (13)	
ABR + R (n = 57)	5 (9)		1 (2)	

*Note*: Data are presented as no. (%). “Recurrent Instability” includes dislocation (requiring manual reduction) and/or subluxation (sensation of instability or apprehension). Bolded values indicate statistical significance (*P* < .05).

ABR, arthroscopic Bankart repair; ABR + R, arthroscopic Bankart repair with remplissage; CHBL, critical humeral bone loss.

Recurrent instability and revision surgery rates of the 99 patients successfully contacted for PROs are reported in the appendix table. Four more patients sustained recurrent instability because last clinical follow‐up and were not captured by EMR review. All 4 of these patients were treated nonoperatively. Rates of recurrent instability and revision surgery did not differ significantly between CHBL and non‐CHBL patients in this cohort.

### Multivariable Analysis of PROs

In a multivariable analysis, recurrent instability was a significant independent predictor of inferior WOSI score and pVAS scores. After adjusting for age, sex, DTD, recurrent instability, remplissage augmentation, and follow‐up time, the presence of CHBL was not a significant independent predictor of WOSI, pVAS, or SSV scores (Table 5).

**TABLE 5:** Multivariable Linear Regression of the Presence of Critical Humeral Bone Loss on Patient‐Reported Outcomes (n = 99)

**Correlate**	**β**	**95% CI**	***P* Value**
WOSI			
CHBL	−85.96	−152.79 to 80.87	.31
Recurrent instability	362.39	195.38 to 529.41	**<.01**
pVAS			
CHBL	−0.37	−1.42 to 0.69	.49
Recurrent instability	1.22	0.16 to 2.27	**.02**
SSV			
CHBL	−3.48	−12.36 to 5.40	.44
Recurrent instability	−3.91	−12.55 to 4.72	.37

*Note*: All models are adjusted for age, sex, distance to dislocation, the presence of recurrent instability, remplissage augmentation, revision surgery, and follow‐up time. Covariates which did not meet statistical significance are not shown here. Bolded values indicate statistical significance (*P* < .05).

CHBL, critical humeral bone loss; CI, confidence interval; pVAS, pain Visual Analog Scale; SSV, Subjective Shoulder Value; WOSI, Western Ontario Shoulder Instability Index.

## DISCUSSION

The most important finding of this study was that patients with CHBL had similar PROs after ABR or ABR + R compared with patients without CHBL. Second, among patients with CHBL, undergoing ABR + R resulted in a significantly lower rate of recurrence, yet similar PROs, compared with patients who underwent isolated ABR. Only recurrent instability was an independent predictor of inferior PROs after adjusting for several covariates. The findings of the present study suggest that patients with CHBL can expect similar subjective outcomes compared with patients without CHBL after ABR or ABR + R at a mean follow‐up of over 7 years, provided they do not incur recurrent shoulder instability.

Most literature on anterior shoulder instability has centered on recurrence rates, yet PROs are increasingly recognized as a critical measure of surgical success. Numerous studies have showed significant improvement in PROs after ABR,^20^, ^21^, ^22^ and several have reported superior PROs with ABR + R compared with ABR alone.^23^, ^24^, ^25^, ^26^ The present study found similar PROs between ABR and ABR + R in patients with CHBL, suggesting that the addition of remplissage may not independently influence long‐term PROs in this specific patient population. Further, the association between recurrent instability and inferior PROs found in this study aligns with existing literature. This study adds to the existing understanding of PROs by revealing that subjective patient outcomes after ABR or ABR + R are multifactorial and not solely dependent on anatomic variables such as CHBL. PROs capture a combination of physical function, pain, confidence, and return to activity, all of which may be influenced by psychosocial factors,^27^, ^28^ rehabilitation,^29^ and patient expectations^30^ as much as by surgical technique or underlying bone morphology. Consequently, while CHBL has been shown to increase the likelihood of recurrent anterior shoulder instability after ABR,^7^, ^11^ its influence on patient perception of shoulder function and satisfaction may be less pronounced.

An unexpected finding of the present study was that rates of recurrent anterior shoulder instability did not differ significantly between CHBL and non‐CHBL cohorts, which contrasts to prior literature revealing higher recurrence rates in patients with CHBL by Cong et al.^7^ Differences in statistical methodology may partially explain this discrepancy because the present study did not adjust for potential confounders such as age, GBL, or participation in contact athletics. Furthermore, Cong et al. included only ABR patients in their analysis, whereas the present study included both ABR and ABR + R. The observed numerical difference in recurrence between cohorts (22% vs 15%) should nonetheless be interpreted with caution because the possibility of a clinically meaningful difference that was not detected cannot be excluded, particularly given the retrospective design and absence of multivariable adjustment for known confounders such as age and DTD, which differed significantly between groups at baseline. However, the current findings align with those of the work of Steuer et al. and Honoki et al., who reported a protective effect of remplissage against recurrent instability in patients with CHBL.^8^, ^9^

An important consideration is that over the period from which patients were retrospectively queried, the indications and popularity of remplissage at our institution changed. Patients included in this study underwent ABR or ABR + R prior to the published findings of Cong et al. and Steuer et al., which established CHBL as a risk factor for recurrent anterior shoulder instability after ABR and the protective effects of ABR + R, respectively.^7^, ^8^ This fact allowed us to evaluate patients who underwent ABR alone and who, based on evidence and our current indications (e.g., the presence of CHBL), would have undergone ABR + R.

The comparable PROs observed across cohorts suggest that all‐arthroscopic techniques for the appropriate indications may provide satisfactory long‐term outcomes even in cases traditionally considered at higher risk of recurrent anterior shoulder instability. These findings serve as an important consideration, given that remplissage remains less commonly performed worldwide despite its increasing popularity in North America. Overall, the results of the present study reveal that PROs after arthroscopic stabilization reflect multiple interrelated factors and CHBL alone may not preclude favorable long‐term results.

### Limitations

This study has several limitations. First, this study is a single‐center, retrospective design, which introduces the potential for selection bias and limits generalizability. Although efforts were made to ensure comparable baseline characteristics between groups, unmeasured factors such as surgeon decision‐making, patient activity level, or rehabilitation adherence may have influenced both treatment selection and outcomes. There were also significant differences in age and DTD values between patients with and without CHBL, which may confound results.

Second, this study relied on voluntary telephone interviews, without concurrent physical examination, to assess PROs. Our PRO response was low (26%) and inherently subject to selection bias. Although validated PRO instruments were used, PROs are subjective and may not fully capture subtle differences in function or instability perception. Most recurrent anterior shoulder instability was ascertained via EMR review, which likely underestimates true instability events. Patients who experienced subluxation or apprehension but did not seek further care would not be captured by this method. Four additional recurrence events were identified only through direct patient contact, none of which had prompted a clinical encounter. Furthermore, we did not have preoperative PRO data, which prevented us from conducting a cohort‐specific MCID analysis.

Based on our power analysis, the CHBL cohort did not meet the required sample size of 54 patients. Although we attempted to contact all consecutive patients who met initial inclusion criteria, this study is at risk of being underpowered to detect a significant difference between the 2 cohorts if the difference is at or below half of the MCID for WOSI. However, this effect size was intentionally chosen to be smaller than a previously published MCID because MCID is not a fixed value and can vary based on the study population. Thus, the study may still be adequately powered to detect a significant difference at or slightly above a previously published MCID.

## CONCLUSIONS

Patients with CHBL had similar long‐term PROs to those without CHBL after ABR or ABR + R at a minimum follow‐up of 1 year. Furthermore, the addition of remplissage was found to be associated with a reduced rate of recurrent anterior shoulder instability in patients with CHBL.

## SUPPORTING INFORMATION

Additional supporting information can be found online in the Supporting Information section.

## DISCLOSURES

The authors (T.C., B.P.L., J.D.H., Albert Lin) declare the following financial interests/personal relationships which may be considered potential competing interests: T.C. reports payment or honoraria for lectures, presentations, speakers bureaus, manuscript writing, or educational events from Smith & Nephew, Mid‐Atlantic Surgical Systems, and Arthrex. B.P.L. reports payment or honoraria for lectures, presentations, speakers bureaus, manuscript writing, or educational events from Mid‐Atlantic Surgical Systems. J.D.H. reports grants or contracts from Arthrex; payment or honoraria for lectures, presentations, speakers bureaus, manuscript writing, or educational events from Mid‐Atlantic Surgical Systems and Smith & Nephew; and other financial or nonfinancial interests from the Editorial Board of *KSSTA* (Stryker) and SI BONE. Albert Lin reports consulting fees from Arthrex, restor3d, and Stryker; payment or honoraria for lectures, presentations, speakers bureaus, manuscript writing, or educational events from Mid‐Atlantic Surgical Systems; support for attending meetings and/or travel from Linvatec; leadership or fiduciary role in other board, society, committee, or advocacy group, paid or unpaid, from restor3d (*Arthroscopy* Journal), ISAKOS (Annals in Joint), ASES (Rotator Cuff Study Group), and *JBJS* Case‐Connector (Arthroscopy Association of North America); and other financial or nonfinancial interests from the Editorial Board of *KSSTA*. The other authors (R.G., R.T.L., S.D., Andrew Liu, A.P., M.R.) declare that they have no known competing financial interests or personal relationships that could have appeared to influence the work reported in this paper.

## ACKNOWLEDGMENTS

The authors would like to acknowledge the following individuals for their assitance in data acquisition and/or analytical expertise: Tyler C. Williams, B.S., Maite Jaspers, M.D., Neel Bharadwaj, B.S., Kyle E. Andrade‐Bucknor, B.S., and Shaquille Charles, M.D.

## Supporting information

Supplementary Material
