# Supplementary material for: Patients With Critical Humeral Bone Loss Have Similar Patient‐Reported Outcomes to Patients Without Critical Humeral Bone Loss After Arthroscopic Shoulder Stabilization at a Mean Follow‐Up of 7 Years
Source: Arthrosc Sports Med Rehabil. 2026 Nov 3:e70140. Online ahead of print. doi: 10.1002/ars2.70140 (PMC13729693; doi:10.1002/ars2.70140)
Supplement: Supplementary file 1 [file ARS2-9999-e70140-s001.pdf]

Appendix

Appendix Table 1. Demographics and Rates of Recurrent Instability in Patients Available for PRO Reporting.

	**Total** (n=99)	**No CHBL** (n=58)	**CHBL** (n=41)	p-value
Age at Surgery (years)	23 ± 9	22 ± 8	25 ± 10	0.10
Male Sex	77 (78%)	45 (78%)	32 (78%)	0.96
Contact Athlete	43 (43%)	26 (45%)	17 (41%)	0.74
Shoulder Hyperlaxity	13 (13%)	5 (9%)	8 (20%)	0.14
% Glenoid Bone Loss	7 ± 7	6 ± 7	8 ± 9	0.26
DTD (mm)	11 ± 6	13 ± 6	9 ± 4	**<0.01**
Procedure				**0.02**
ABR	68 (69%)	45 (78%)	23 (56%)	
ABR+R	31 (31%)	12 (21%)	18 (44%)	
Recurrent Instability	19 (19%)	10 (17%)	9 (22%)	0.61
Revision Stabilization	4 (4%)	4 (7%)	0 (0%)	0.14

Note: Data are presented as mean ± SD for continuous measures, and No. (%) for categorical measures. Data are significant if p<0.05. ABR = Arthroscopic Bankart Repair. ABR+R = Arthroscopic Bankart Repair with Remplissage. CHBL = Critical Humeral Bone Loss. DTD = Distance to Dislocation.

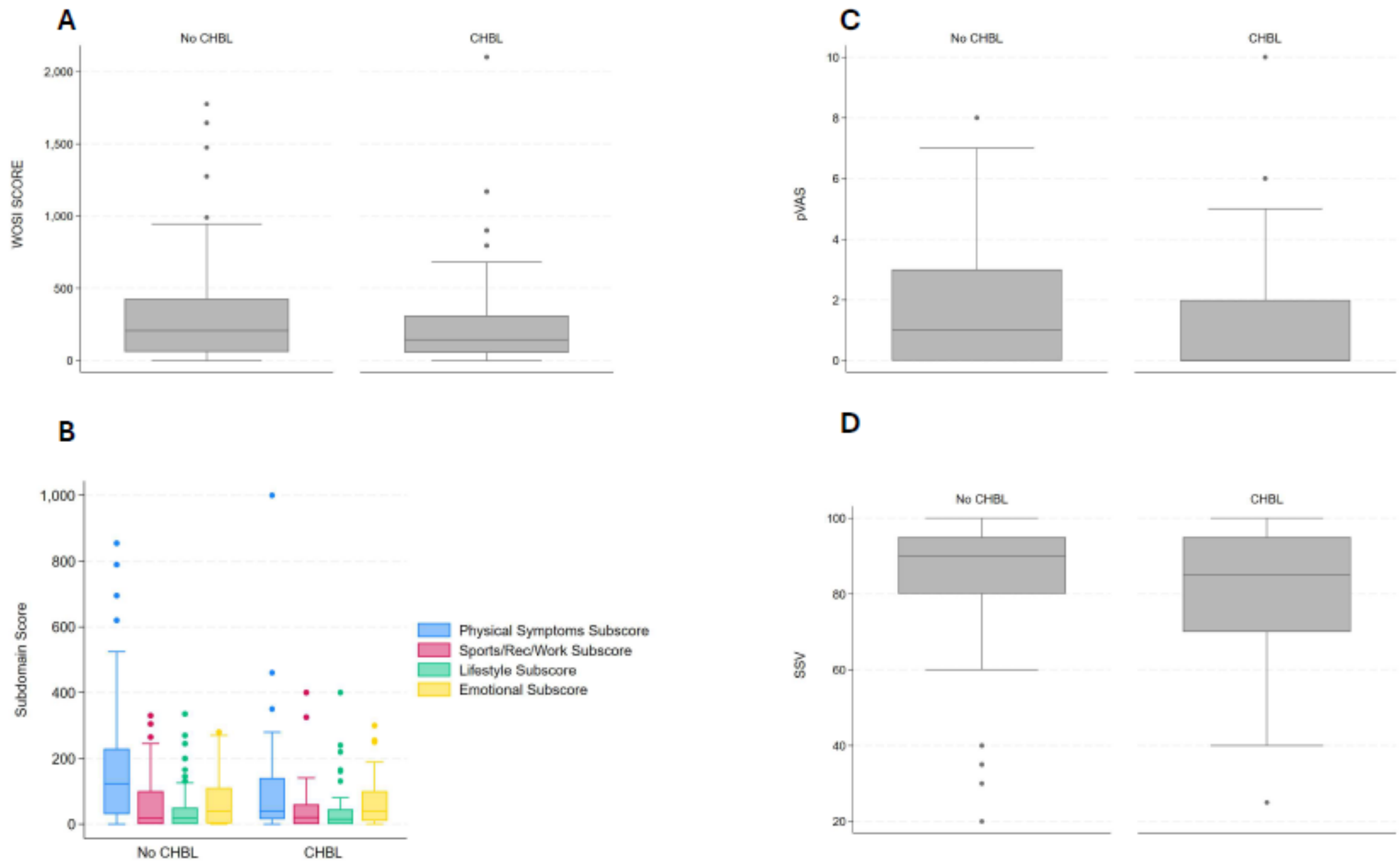

Appendix: Patient Reported Outcomes, Stratified by Critical Humeral Bone Loss. No significant differences were identified in any of the patient-reported outcomes at final follow up between patients with critical humeral bone loss and those without critical humeral bone loss. **A.** Western Ontario Shoulder Stability Index (WOSI). **B.** The four subdomains of the WOSI score. **C.** The Visual Analog Scale for Pain (pVAS). **D.** The Subjective Shoulder Value (SSV).

CHBL = Critical Humeral Bone Loss
